# Snake Venom Pharmacokinetics and Acute Toxic Outcomes Following *Daboia siamensis* Envenoming: Experimental and Clinical Correlations

**DOI:** 10.3390/toxins17010010

**Published:** 2024-12-29

**Authors:** Sethapong Lertsakulbunlue, Wipapan Khimmaktong, Orawan Khow, Wittawat Chantkran, Jureeporn Noiphrom, Kanyanat Promruangreang, Lawan Chanhome, Janeyuth Chaisakul

**Affiliations:** 1 Department of Pharmacology, Phramongkutklao College of Medicine, Bangkok 10400, Thailand; sethapong.ler@pcm.ac.th; 2 Division of Health and Applied Sciences, Faculty of Science, Prince of Songkla University, Songkhla 90110, Thailand; wipapan.k@psu.ac.th; 3 Research and Development, Queen Saovabha Memorial Institute, Thai Red Cross Society, Bangkok 10330, Thailand; okhow_2000@yahoo.com (O.K.); tu-juree-n@hotmail.com (J.N.); 4 Department of Pathology, Phramongkutklao College of Medicine, Bangkok 10400, Thailand; chantkran@gmail.com; 5 Forensic Toxicology Unit, Department of Forensic Medicine, King Chulalongkorn Memorial Hospital, Bangkok 10330, Thailand; kanyanat_prom@yahoo.com; 6 Snake Farm, Queen Saovabha Memorial Institute, Thai Red Cross Society, Bangkok 10330, Thailand; lchanhome@yahoo.com

**Keywords:** venom, Russell’s viper, histopathology, envenoming, snakebite

## Abstract

An understanding of snake venom pharmacokinetics is essential for determining clinical outcomes of envenoming and developing therapeutic approaches to the treatment of envenoming, especially regarding the timing and optimal dosage of antivenom administration. *Daboia siamensis* (Eastern Russell’s viper) envenoming causes systemic coagulopathy and severe hemorrhage including acute kidney injury. These toxic outcomes can be diminished by the administration of high quantities of Russell’s viper antivenom. This study aimed to determine the correlation between the clinical profiles of *D. siamensis* envenomed patients and experimental data by measuring plasma venom concentration and conducting histopathological analyses of heart, kidney, and liver tissues in rats 6 h after experimental *D. siamensis* envenomation. Intramuscular (i.m.) administration of *D. siamensis* venom to anesthetized rats (200 µg/kg) resulted in a rapid absorption of venom which reached a peak concentration at 60 min before declining and then plateauing. Urine samples detected 209.3 ± 21.6 ng/mL of *D. siamensis* venom following i.m. administration at 6 h. Histopathological studies showed morphological changes in heart, kidney, and liver tissues following 3 h experimental envenoming and exhibited a higher degree of severity at 6 h. A retrospective study of the clinical profile and laboratory examination of Russell’s viper envenomed patients in Central Thailand was also evaluated, showing that systemic coagulopathy and local effects were commonly observed in the early stage of *D. siamensis* envenoming. An abnormal increase in creatinine levels was found in 13.6% of the population. Early administration of specific antivenom within 1–2 h following envenoming is highly recommended to prevent life-threatening outcomes such as severe coagulation and acute kidney injury.

## 1. Introduction

Snakebite is a significant occupational hazard in many rural areas of South America, Sub-Saharah Africa, South Asia, and Southeast Asia [1]. In 2017, the World Health Organization (WHO) re-recognized snakebite envenoming as a neglected tropical disease with an estimated 1.8–2.7 million snakebite cases each year [2]. Of these victims, there are around 81,000–138,000 deaths [2,3]. Snakes use venom to facilitate prey capture and as a defensive mechanism against predators. Snake venom is a complex mixture of enzymatic and non-enzymatic components capable of causing a significant number of clinical outcomes, such as skeletal muscle paralysis leading to respiratory failure, excessive bleeding, or venom-induced consumption coagulopathy including severe tissue damage resulting in amputation of affected organs [4].

Russell’s vipers (*Daboia* spp.) are one of the most medically significant venomous snake genera in Asia [4]. The Eastern Russell’s viper (*D. siamensis*), which is categorized as a true viper (subfamily Viperinae), causes high mortality and morbidity rates (category 1) in Myanmar, Thailand, and some Indonesian islands, specifically in Java, Komodo, Flores, and Lomblen [4]. In addition, the distribution of Eastern Russell’s viper (*D. siamensis*) also includes South China (Guangdong and Guangxi) and the insular Taiwan [5]. Acute kidney injury (AKI) and disseminated intravascular coagulopathy are commonly observed following envenoming by *D. siamensis* [6,7,8,9,10]. Interestingly, *D. siamensis* venom was recently reported to induce neurotoxicity, causing inhibition of the chick biventer cervicis nerve-muscle preparation [11]. A number of studies have reported the basic proteomic profile of *D. siamensis* venom which is comprised of phospholipase A_2_ (PLA_2_), snake venom metalloproteinases (SVMP), flavin monoamine oxidase, and serine protease toxin families [12,13]. Other toxic components (e.g., L-amino acid oxidases (LAAO), phosphodiesterase (PDE) and C-type lectin-like proteins [14]) also contribute to clinical outcomes following envenoming by *Daboia* spp. Indeed, the purified PLA_2_ and SVMP from *D. siamensis* venom have been demonstrated to initiate kidney injury via an increase in renal vascular resistance or renal ischemia, resulting in decreases in renal blood flow, glomerular filtration rate, and urine flow [15,16].

Many studies have examined the pharmacokinetics or toxicokinetics of snake venom in animal models and envenomed patients in order to demonstrate the time course of toxicity concerning venom concentrations and identify the optimum period of antivenom administration [17,18,19,20]. However, a previous study of Russell’s viper venom pharmacokinetics in envenomed patients showed that the venom levels did not correlate with the time of venom injection, which may indicate the release of venom from the depot at the bite site [21].

Moreover, the investigation of toxicity or venom pharmacodynamics following experimental snake envenoming has also been performed in animal models in correlation with measuring blood pressure and heart rate to monitor cardiovascular responses [22], or the determination of serum creatinine and blood urine nitrogen (BUN) levels to investigate snake-venom-induced myotoxicity and nephrotoxicity [18,23]. In addition, histopathological changes in skeletal muscle and kidney tissue are used to determine the pathology following myotoxic or nephrotoxic snake envenoming, respectively [23,24].

As envenoming by Russell’s viper may cause significant morbidity and mortality, the precision of diagnosis or prediction of clinical outcomes using the correlation between circulating snake venom concentration and early clinical manifestation would help to reduce the mortality rate or to prevent serious complications following *D. siamensis* envenoming. Therefore, this study determined the association between *D. siamensis* venom pharmacokinetics in an anesthetized rat model and histopathological changes in heart, kidney, and liver tissues following the experimental *D. siamensis* envenoming in the acute stage. We also examined the correlation of experimental data with a retrospective Russell’s viper-bitten patient profile from central Thailand. The knowledge from this study will provide important insights into the effective clinical approach for snake envenomed victims.

## 2. Results

### 2.1. Clinical Profile of Russell’s Viper Bite Patients

A cross-sectional study was conducted to determine the prevalence and management of Russell’s viper bite patients at Pattananikom Hospital, Lopburi Province.

#### 2.1.1. Demographic Characteristics

Between 2014 and 2023, a total of 23 patients with Russell’s viper bites visited Pattananikom Hospital in Central Thailand. The demographic characteristics of the victims are shown in Table 1. Males accounted for 82.6% of all patients. The time from the bite to arrival at the emergency department ranged from 10 to 90 min. Most victims had been bitten on the lower extremities (73.9%). According to physical examination and subjective examination, Russell’s viper-bitten patients had local pain (65.2%), erythema (87%), swelling at the bite site (78.3%), and dyspnea.

#### 2.1.2. Laboratory Investigation Results of Patients with *D. siamensis* Bites

In Table 2, the majority of patients had normal levels of serum sodium (78.3%), serum creatinine (86.4%), bicarbonate (65.2%), and white blood cell count (82.6%). However, hypokalemia appeared to occur in more than half of victims following a *D. siamensis* bite (65.2%). Laboratory data in Table 2 also indicated hyponatremia (21.7%), metabolic acidosis (34.8%), and a high level of white blood cells (17.4%).

#### 2.1.3. Hematological and Systemic Effects of Patients Following *D. siamensis* Bites

Systemic bleeding was found in most cases (82.6%). Data of hematological examinations were performed within 6 h after the hospital visit. No prolonged venous clotting time was observed. However, 26.1% of Russell’s viper bite patients had a prolonged international normalized ratio (INR). Low levels of platelets occurred in around 22% of patients. Administrations of *D. siamensis* antivenom were performed in 43.5% of envenomed patients (Table 3).

### 2.2. Measuring of Venom Concentration in Experimentally Envenomed Rats

Determination of *D. siamensis* venom concentration was performed using the ELISA technique, as described in the methodology section.

#### 2.2.1. Serum Venom Concentration

**Figure 1:**
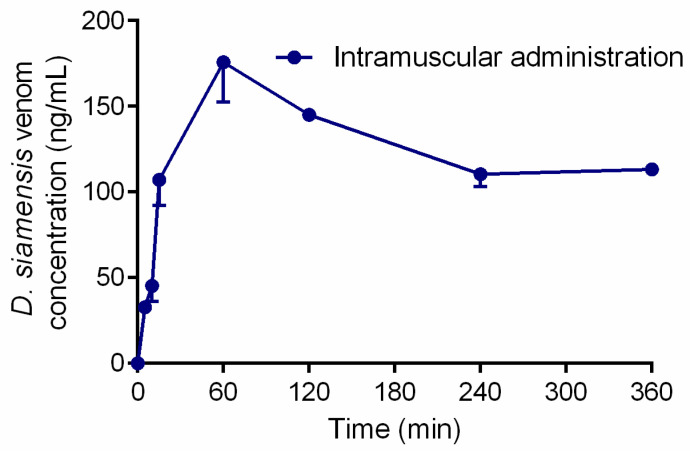
*D. siamensis* venom concentration following i.m. administration for 360 min (*n* = 5).

The time course of the serum venom concentrations after administration of *D. siamensis* venom (200 µg/kg, i.m.) is shown in Figure 1. Venom injection via the intramuscular route caused an initial rapid rise in venom concentrations (absorption) to a peak value of approximately 170 ng/mL at 60 min. A plateau in the serum venom concentration was observed at approximately 120 ng/mL from 240 min to the last time point of sample collection of 360 min.

#### 2.2.2. Urine Venom Concentration

Urine samples from experimentally envenomed rats were collected at 60, 120, and 360 min following i.m. administration of *D. siamensis* venom. *D. siamensis* venom levels were 35.9 ± 9.9 ng/mL (*n* = 5) and 42.9 ± 8.4 ng/mL ng/mL (*n* = 5) at 60 and 120 min, respectively. After 360 min, a urine venom concentration of 209.3 ± 21.6 ng/mL (*n* = 5) was detected.

### 2.3. Histopathological Study of Heart, Kidney, and Liver Tissues Following D. siamensis Envenoming

#### 2.3.1. Morphological Changes in Heart Following Envenoming

**Figure 2:**
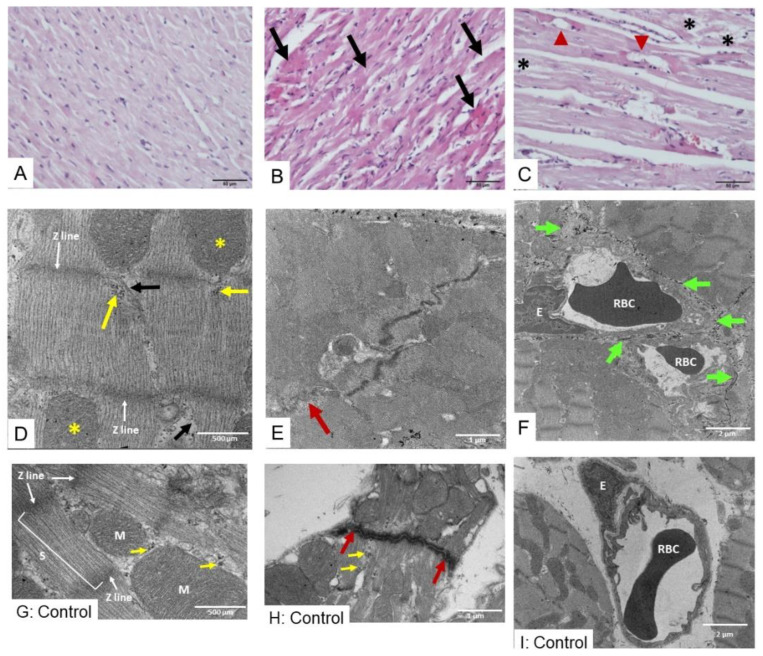
(**A**) Histopathological changes in heart tissue in H&E staining image following i.m. administration of saline in rats (scale bar = 50 µm). Intramuscular administration of *D. siamensis* venom dose 200 µg/kg (scale bar = 50 µm) for 3 h caused (**B**) muscle fiber hypertrophy (black arrows) and irregular shape and (**C**) dilatation of blood vessels (red triangles), aggregating of red blood cells, and loss of myofibrils (black asterisks) in some areas (scale bar = 50 µm). The histopathological determination of heart tissue under TEM indicates that administration of *D. siamensis* venom for 3 h caused (**D**) disarrangement and disappearance of myofibrils (black arrow; scale bar = 500 µm), as well as sarcomeres and Z-line disruption and swollen mitochondria (yellow asterisk) including (**E**) partially organized gap junction (red arrow) at area of intercalated discs between cardiac myofibrils (scale bar = 1 µm). Administration of *D. siamensis* venom (200 µg/kg) also caused (**F**) the appearance of the collagen fiber fragments (green arrows; scale bar = 2 µm). TEM examination of heart tissue following administration of saline shows (**G**) myofibril with sarcomere and intact mitochondria (scale bar = 500 µm), (**H**) intact gap junction (red arrows; scale bar = 1 µm)), and (**I**) intact capillary between cardiac myofibrils and endothelial cells (scale bar = 2 µm). Yellow arrows indicate glycogen particles. **M** indicates mitochondria. **S** indicates sarcomere. **E** indicates endothelial cells. **RBC** indicates red blood cells.

Administration of saline (i.m.) caused undetectable histopathological changes in heart tissue (Figure 2A) with the intact myofibril and normal shape of mitochondria and capillary (Figure 2G–I). Three hours after administering *D. siamensis* venom, minor injury of cardiac muscle fibers and a few inflammatory cells (Figure 2B,C) were observed. The absence of cardiac myofibrils and mitochondrial swelling (Figure 2D) were found under TEM (Figure 2D), including a partially organized gap junction (red arrow) at the area of intercalated discs between cardiac myofibrils (Figure 2E) and the appearance of collagen fiber (Figure 2F).

**Figure 3:**
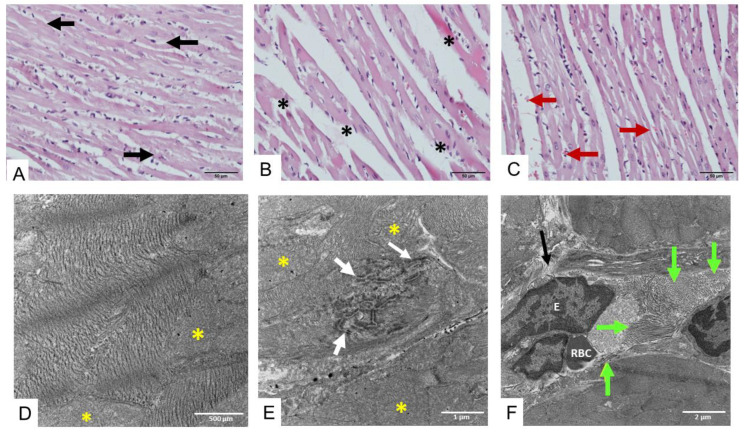
Histopathological examination of heart tissue following 6 h intramuscular administration of *D. siamensis* venom (200 µg/kg) in rats showing (**A**) irregular shape of muscle fibers (black arrows; scale bar = 50 µm) including (**B**) torn muscle fibers and loss of myofibrils (black asterisks). H&E staining also exhibits (**C**) the red blood cells (red arrows) distributed between 6 h envenomed tissue muscle fibers. The histopathological determination of heart tissue under TEM indicates that administration of *D. siamensis* venom causes (**D**) swollen mitochondria (yellow asterisk) and (**E**) disarranged intercalated discs between myofibrils (white arrows) throughout the tissue, including (**F**) the appearance of collagen fiber slips into the lumen of the blood vessel (green arrows) and separation of the blood vessel wall (black arrow). **E** indicates endothelial cells. **RBC** indicates red blood cells.

Administration of *D. siamensis* venom (200 µg/kg, i.m.) for 6 h caused a high degree of injury of cardiac muscle fiber, swollen myofibrils, and the presence of blood clotting between cardiac muscle fibers (Figure 3A–C). The histopathological determination of heart tissue under TEM indicates that administration of *D. siamensis* venom for 6 h induced swollen mitochondria (Figure 3D) and disarranged intercalated discs between myofibrils throughout the tissue (Figure 3E), including the appearance of collagen fiber slips into the lumen of the blood vessel and separation of the blood vessel wall (Figure 3F).

#### 2.3.2. Morphological Changes in the Kidney Following Envenoming

**Figure 4:**
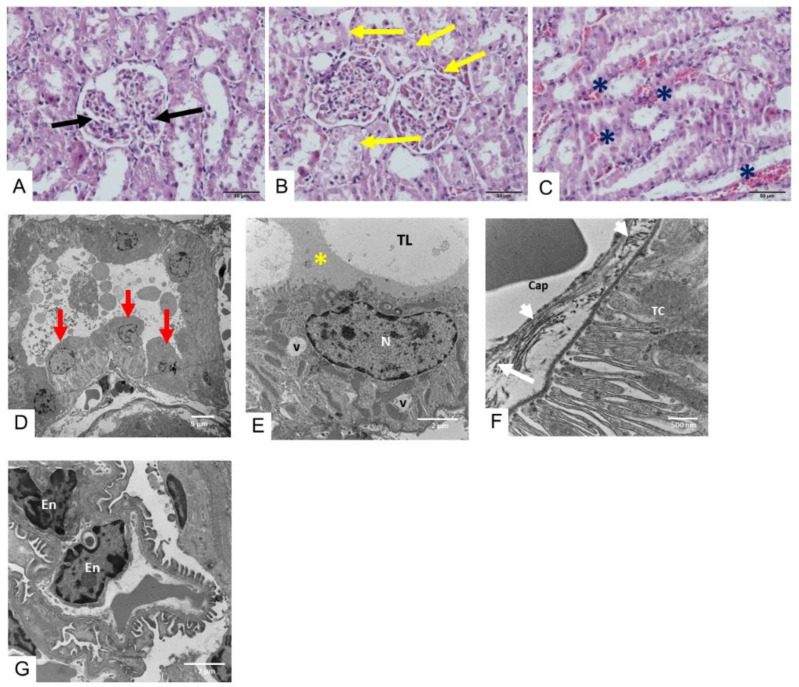
(**A**) Histopathological changes in kidney tissues of rats (H&E staining; scale bar = 50 µm) following i.m. administration of saline showing (**A**) glomerular cells (black arrows; scale bar = 50 µm). Histopathological changes (H&E staining) in kidney tissues of rats following 3 h i.m. administration of *D. siamensis* venom (200 µg/kg) showing (**B**) mild tubular necrosis (yellow arrows; scale bar = 5 µm) and (**C**) detectable blood clots (asterisks). The histopathological determination of kidney tissue under TEM shows that i.m. administration of *D. siamensis* venom (200 µg/kg) for 3 h causes (**D**) degenerated tubular epithelial cells (red arrows, scale bar = 5 µm). (**E**) At high magnification (scale bar = 2 µm), a distal tubule cell with many cytoplasmic vesicles (V) is involved in the reabsorption of glycoprotein cast (yellow asterisk). The nucleus (N) of the distal tubule is present in most cells and lies close to the tubular lumen (TL). (**F**) A medium amount of collagen fiber (white arrows) was found at the wall and the area around the capillary (Cap). TC indicates tubular cell (**G**) The effacement of podocyte foot processes is exhibited, while the glomerular basement membrane structure is normal in the endothelial cells (En) of the glomerular capillary.

Administration of saline did not cause any change in the histopathological appearance of the kidney under light microscopy (Figure 4A) and TEM. Following administration of *D. siamensis* venom (200 µg/kg, i.m.) for 3 h, mild tubular necrosis (Figure 4B), a detectable blood clot (Figure 4C) and degenerated epithelial cells (Figure 4D), including glycoprotein cast (Figure 4E), were observed. In addition, we also found a medium amount of collagen fiber (white arrows) at the wall and at the area around the capillary (Figure 4F), while the endothelial cells of the glomerular capillary were still intact (Figure 4G).

**Figure 5:**
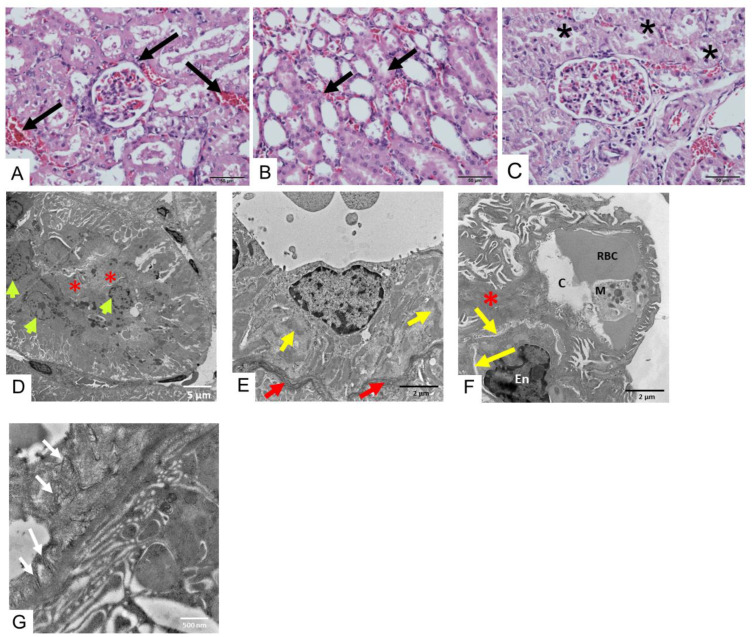
Histopathological examination of kidney tissue (H&E staining) following 6 h intramuscular administration of *D. siamensis* venom (200 µg/kg) in rats shows (**A**) aggregation of red blood cells in the glomerulus and (**B**) renal tissue (black arrows; scale bar = 50 µm) including (**C**) tubular epithelial cell necrosis (asterisks). TEM determination shows (**D**) the electron-dense cast in distal tubules (red asterisk) and epithelium necrosis (green arrows). (**E**) At high magnification, tubular epithelial cells showed degenerate mitochondria (yellow arrows) and tubular basement epithelium (red arrows). (**F**) Severe injury of capillary loops in the glomerulus was observed. The edematous endothelial cell had detached from the glomerular basement membrane (yellow arrowheads). The necrotic capillary loop was filled with a macrophage. Flattened foot processes of podocytes show densely packed (red asterisk). M = macrophage, En = endothelial cell, C = capillary lumen.; RBC = red blood cells. (**G**) A small amount of collagen fiber (white arrows) was found around the capillary wall.

Administration of *D. siamensis* venom (200 µg/kg, i.m.) in rats for 6 h induced aggregation of red blood cells in the glomerulus and renal tissue (Figure 5A,B), including renal tube necrosis (Figure 5C). Under TEM determination, there was an electron-dense cast in distal tubules and epithelium necrosis (Figure 5D). At high magnification, tubular epithelial cells showed degenerate mitochondria and tubular basement epithelium (Figure 5E). Severe injury of capillary loops in the glomerulus was observed. The edematous endothelial cell had detached from the glomerular basement membrane (Figure 5F). A small amount of collagen fiber (white arrows) was found around the capillary wall (Figure 5G).

#### 2.3.3. Morphological Changes in the Liver Following Envenoming

**Figure 6:**
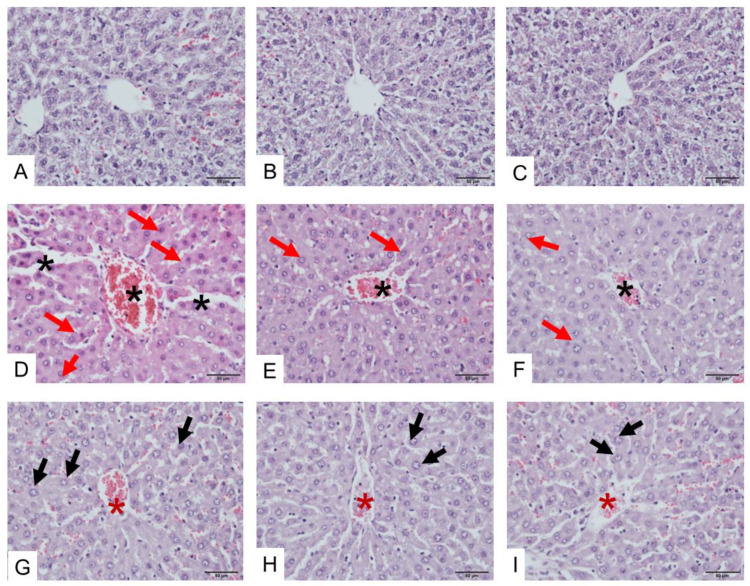
Histopathological examination of liver tissue (H&E staining) following the i.m. administration of saline (**A**–**C**) in the comparison with liver tissues following administration of *D. siamensis* venom for 3 h (**D**–**F**) shows a severe widening of central veins and sinusoids (black asterisks) together with the accumulation of red blood cells, as well as swollen and hypertrophic hepatocytes (red arrows). The administration of *D. siamensis* venom for 6 h (**G**–**I**) causes a moderate amount of hepatocyte swelling (black arrows), as well as a moderate widening of central veins (red asterisks) and sinusoids with an aggregation of red blood cells.

The administration of saline did not affect liver tissues either in the portal triad or central vein (Figure 6A–C). Administration of *D. siamensis* venom (200 µg/kg, i.m.) for 3 h caused partial inflammation of hepatocytes around the portal triad, congestion of sinusoids, diffused hepatic necrosis, amyloidosis (Figure 6D), edema of hepatocyte cells (Figure 6E), and congestion of the central vein (Figure 6F). The administration of venom for 6 h induced a high degree of inflammatory cells with lymphocytes and sinusoids with an aggregation of red blood cells (Figure 6G–I).

## 3. Discussion

A more thorough understanding of snake venom pharmacokinetics would be beneficial for optimizing treatment following snake envenoming, including the timing and dosage of antivenom. There are a number of factors that contribute to snake venom pharmacokinetics, including the molecular weight of various toxins, the snake species responsible for the bite, and the route of administration of the venom [25]. The size and molecular weight of venom components may cause differences in absorption [26]. In addition, the length of the snake fangs is also an important determinant in whether the venom is injected subcutaneously (short-fanged elapid) or intramuscularly (long-fanged viperid) [26].

### 3.1. Snake Venom Pharmacokinetic Study in Animal Model

In the present study, *D. siamensis* venom pharmacokinetics was explored in order to determine a correlation between venom levels and clinical outcomes following Russell’s viper envenoming within 6 h. Enzyme-linked immunosorbent assays (ELISA) are widely used to measure venom concentration in studies of snake venom pharmacokinetics [18,19,27]. However, limitations of this technique have been reported, including cross-reactivity against the venoms of closely related snake species and the limit of detection for snake venoms and toxins between 0.1 and 20 µg/L, including a long incubation time of the antibody [28,29]. In order to avoid these limitations, researchers have attempted to develop specific and rapid detection tools for detecting snake species and measuring snake venom concentration for the clinical setting. One such technique utilizes a biosensor, which could be used to measure venom concentration in plasma by using the electrochemical activity of the coated antibody and the venom in plasma [30].

The initial phase of venom pharmacokinetics was reported to represent the distribution, and the elimination of venom occurred in the termination phase [25]. The plasma concentration–time course following i.m. administration of *D. siamensis* venom showed a rapid absorption of the venom in central circulation until approximately 60 min post-venom administration. After this time, we observed a slight decrease in venom concentration, resulting in a plateau. This pattern of concentration–time profile has been postulated to involve some processes during absorption and distribution, such as absorption via lymphatic circulation [31] or binding to some targets in the central compartment [18,32]. Many studies examined the pharmacokinetics of snake venom following i.m. administration versus i.v. administration [17,19,27], and found that not all venom or toxins were fully absorbed into the circulation following i.m. administration. Different venom administration routes (i.m. vs. i.v.) can cause variations in the duration of snake venom half-life. The prolonged duration of the half-life was also mentioned to be the effect of a prolonged absorption period, as the disposition processes should not be affected by administration processes [25]. Moreover, the release of venom from a depot at the bite site has been suggested to affect the kinetic parameter of venom concentration during the distribution step in envenomed patients [21].

Similar to a previous study using a rat model to examine myotoxicity following mulga snake (*Pseudechis australis*) envenoming [18], we also had some limitations for analyzing pharmacokinetics in the early period of snake envenoming (i.e., within 6 h). In this study, there were insufficient data points to evaluate the elimination phase which required 6–12 h due to the small amount of blood and urine samples that can be collected from a rat.

### 3.2. Toxic Outcomes Following D. siamensis Envenoming in Experimentally Envenomed Animals

Snake-venom-induced consumption coagulopathy and acute kidney injury are commonly observed following *D. siamensis* envenoming. In this study, experimentally envenomed rats were sacrificed at 3 and 6 h following i.m. administration of *D. siamensis* venom for histopathological determination of heart, kidney, and liver tissues. At 3 h after envenoming, partial injuries were found in all collected tissues. Inflammation of cardiac muscle fiber and swelling of mitochondria of cardiac myocytes were detected under TEM. It has been reported that there are increases in leukocytes, cyclooxygenase-2, and eicosanoids related to local inflammation following the intraperitoneal administration of purified PLA_2_ of *D. siamensis* venom to the mice for 2 h [33], suggesting that venom or toxins in the systemic circulation can rapidly induce the generation of inflammatory mediators during the distribution period (i.e., 30 min to 2 h). This is the first histopathological study to show early morphological changes in tissue following experimental envenoming of *D. siamensis* venom at 3 or 6 h, as our previous studies demonstrated histopathological changes in tissues 12 or 24 h after venom administration [12,23].

Nephrotoxicity observed following *D. siamensis* envenoming, i.e., acute kidney injury (AKI), was shown to be generated by several toxic components in the venom, such as RvPLA_2_, RvMP, RvLAAO, and RvPDE. These toxins caused a decrease in the glomerular filtration rate, urinary flow rate, and osmolar clearance [34]. Previously, we showed significant increases in BUN (blood urea nitrogen) and creatinine levels following i.m. administration of *D. siamensis* venom for 3 to 6 h [23] which support our current histopathological evaluation where morphological changes in the kidney were detected 3 h after experimental envenoming. Moreover, a study conducted by Mello and colleagues (2010) demonstrated that immunohistochemistry detected *Bothrops alternatus* venom in glomeruli, proximal, and distal tubules, as well as vascular tissues, at 3 and 6 h following experimental envenomation [35]. This finding suggests that the pharmacokinetic distribution of snake venom occurs within 3 h and is associated with nephrotoxicity.

Renal excretion is a critical pathway for the elimination of snake venoms, antivenoms, and their metabolites. Numerous studies have demonstrated the effectiveness of the ELISA technique in detecting snake venom in urine within 1 to 24 h after envenomation [35,36]. Oliguria has also been reported in experimental viper envenomation in rats [34,35]. In the current study, urine samples were collected post-venom administration at 1, 2, and 6 h. A decrease in urine volume was observed during the 2 h sample collection, which limited the ability to collect another sample until 6 h post-administration. The highest venom concentration was detected in the urine at 6 h following i.m. administration, correlating with the severity of kidney damage observed in the histopathological analysis. It has been noted that the presence of venom in renal tissue does not necessarily correlate with its excretion by the kidneys but may contribute to venom-induced nephrotoxicity [35,37].

Morphological changes in liver tissues were found at 3 and 6 h post-venom administration. In fact, elevated ALT and AST levels were reported in Russell’s viper (*D. ruselii*)-envenomed dogs, which could recover up to 120 h [38].

### 3.3. Clinical Observations and Laboratory Examinations of D. siamensis Envenomed Patients

We also performed a retrospective study using data from the standardized case report form of snake-envenomed patients in Thailand’s central region to evaluate and identify an association between clinical laboratory investigation parameters in envenomed patients and laboratory studies. We evaluated clinical and laboratory data of 23 Eastern Russell’s viper bite patients who enrolled in Pattananikom Hospital in Lopburi Province. All laboratory data were obtained and recorded within 6 h of admission at the emergency department. We found that 19 patients were reported to have systemic bleeding (82.6%), 6 patients had INR > 1.2 (26.1%), and 21.7% presented thrombocytopenia. Local effects, including pain at the bite site, swelling, and erythema, occurred in more than 60% of the patients. INR is considered to be the most useful diagnostic test in VICC [39]. Additionally, the present study found that the venous clotting time (VCT) was normal in all cases, aligning with its known lower sensitivity than other tests used to diagnose VICC. Nevertheless, VCT remains a valuable tool due to its ability to be performed at the bedside and provide timely results [39]. No evidence of compartment syndrome and rhabdomyolysis involving acute kidney injury were reported.

Snake antivenom contains polyclonal antibodies purified from immunized animal serum or plasma and remains an effective therapy for Russell’s viper envenoming. According to the guidelines for snakebite-envenomed treatment used across Southeast Asia [4], there are 6 indications for hematotoxic snake antivenom administration, including (1) VCT > 20 min, (2) 20 min whole blood clotting time (20WBCT), (3) INR > 1.2, (4) platelet count < 50 × 10^3^/µL, (5) systemic bleeding, and (6) impending compartment syndrome [4]. In this patient group, some victims (56.5%) did not require antivenom treatment. Administration of *D. siamensis* antivenom was implemented in 10 Russell’s viper-bitten patients (43.5%). An amount of antivenom between three and five vials was required to be administered to the Russell’s viper-envenomed patients. However, eight vials of Russell’s viper antivenom were required to relieve the local and systemic effects of Taiwanese *D. siamensis* envenoming [40]. Interestingly, an envenomed victim with an indication of antivenom treatment (INR = 1.3) did not receive snake antivenom treatment in our current study. Lack of snake antivenom availability, including misuse and inappropriate snake antivenom administration, appears to be problematic in some rural areas of tropical countries. This might be due to poor antivenom stock management and inexperienced practices of the physician [41]. Recently, a retrospective study in southernmost Thailand reported that local bleeding and mild to moderate thrombocytopenia appeared to be the independent factors for inappropriate use of hematotoxic snake antivenom in Malayan pit viper (*Calloselasma rhodostoma*) envenoming [42]. In terms of pharmacokinetic correlation, Hart et al. (2014) demonstrated that administering snake antivenom within hours of venom absorption partially prevented myotoxicity following *Pseudechis australis* envenomation, with earlier antivenom administration providing a greater preventive effect [18].

Other abnormal laboratory parameters in this work, i.e., hyponatremia (21.7%) and metabolic acidosis (34.8%), were found in the minority population, whereas hypokalemia occurred in 65.2% of victims. A previous study reported hyponatremia in Malayan krait-envenomed patients, suggesting the presence of natriuretic peptides in krait venom [43]. Hypokalemia associated with metabolic acidosis was previously reported following common krait (*Bungarus caeruleus*) envenoming, which could be involved in the internal shift of potassium into the cells due to β-adrenergic stimulation as a result of autonomic dysfunction [44]. In addition, leukocytosis with neutrophilia is a stress response commonly found in snakebite envenoming [45]. In this study, leukocytosis occurred in four patients with a white blood cell count over 11 × 10^3^ cell/µL.

### 3.4. Association Between Experimental Study Using Animal Model and Clinical Study

Although histopathological lesions of the kidney were detected in envenomed rat tissue, only 13.6% of the participants exhibited elevated creatinine levels, which is relatively low compared to a previous study [46]. This discrepancy is likely to involve the variation in snake venom composition, route of administration, amount of venom injected by each snake, and biotransformation of snake venom in each patient [18,19]. Moreover, this lack of findings may be attributed to the single time point of clinical laboratory collection and the timely administration of antivenom.

AKI is known to correlate significantly with blood venom concentrations, suggesting that higher venom loads are associated with more severe kidney injury [46]. Therefore, the timely administration of antivenom may mitigate the risk of AKI. Furthermore, AKI can present after eight hours post-envenomation, emphasizing the need for repeated renal function tests to monitor patient prognosis following Russell’s viper envenomation [42,46].

Dyspnea was complained of by one victim, which is very rare in *D. siamensis* envenoming. However, our previous study demonstrated that the purified PLA_2_ from *D. siamensis* venom caused a decrease in skeletal muscle contraction and also induced morphological changes in heart tissue [12]. A number of studies showed that the severity and onset of neurotoxicity and myotoxicity in envenomed victims is dependent on the time after envenoming and the concentration of responsible toxic components in the venom [12,18,23].

Swelling (78.3%) and erythema (87.0%) were clinically recorded at ED in early stage of envenoming, which fell into the absorption period of snake venom. However, data regarding swelling measurement and grading of erythema were unavailable due to the limitation of a retrospective study. In animal studies, i.m. administration of *D. siamensis* envenoming caused significant swelling at the injected hindlimb compared with the intact hindlimb. Measuring the degree of swelling is required in future studies.

### 3.5. Limitations

The present study has several limitations that should be acknowledged. First, the cross-sectional design of the epidemiological study limits the ability to evaluate longitudinal changes and long-term outcomes after discharge. Second, the study’s retrospective nature and its ten-year duration may have resulted in missing data due to incomplete medical records. Third, the study was conducted at a single hospital, limiting the findings’ generalizability to other regions. Moreover, the referral of severe cases to larger hospitals might have led to underestimating the mortality rate. Fourth, the single examination of serum creatinine may lead to a plausible underestimation of AKI. Therefore, caution is advised when interpreting and applying these findings to broader contexts. Lastly, some biomarkers were unavailable in the rat model, and pathological data were unavailable for the patients, preventing direct comparisons between the two.

In addition, the observational time points between the rats and patients may restrictively correspond, making it challenging to directly link venom absorption patterns to early clinical symptoms. Therefore, future studies should consider designing research that establishes a correlation between clinical findings and experimental results in rats.

## 4. Conclusions

In conclusion, the current study shows the association between *D. siamensis* venom concentration and toxic outcomes over 6 h following envenoming in both an experimental model and in a clinical setting. The administration of *D. siamensis* venom to the animals was able to cause detectable lesions in various vital organs after 3 h, which fit in the distribution phase of venom kinetics. In comparison, the elimination phase was also demonstrated by the presence of venom in urine collected at 6 h after envenoming. Our clinical retrospective study in envenomed patients showed that systemic coagulopathy and local effects were observed during the early stage of *D. siamensis* envenoming, especially in 1–2 h.

Although the correlations between clinical and experimental studies were not completely demonstrated due to a number of limitations, this study shows that toxic outcomes observed following early Russell’s viper envenoming could be worsened if an unprecise diagnosis and treatment were provided. Apart from better determining a sufficient dose and timing for snake antivenom treatment, understanding snake venom pharmacokinetics will assist clinicians in predicting the clinical prognosis of the envenomed patients during pre- or post-hospitalization.

## 5. Materials and Methods

### 5.1. Clinical Profile of Envenomed Patients

#### 5.1.1. Study Design and Subjects

Data of Russell’s viper bite victims were collected from 1 October 2014 to 30 August 2023 at Pattananikom Hospital, Lopburi Province.

#### 5.1.2. Data Collection

Data were collected from medical records using a standardized case record form, including (1) participants’ baseline characteristics such as age, gender, visit date, time of arrival at the emergency department (ED), time of snakebite, bite site, and pre-hospital management; (2) laboratory tests conducted at the ED within 3 h before any treatment, including complete blood count (CBC), serum electrolytes, serum creatinine, 20WBCT, VCT, and INR (follow-up records for 20WBCT and VCT were also included); and (3) information on management and outcomes, such as clinical manifestations, admission time, and antivenom treatment. Snakebite cases were identified using the International Classification of Diseases, Tenth Revision (ICD-10) code T63.0, as documented in the medical records [41]. The appropriateness of antivenom administration was also reviewed and extracted from these records.

#### 5.1.3. Ethics Considerations

The Institutional Review Board of the Royal Thai Army Medical Department, conforming to international guidelines such as the Declaration of Helsinki, the Belmont Report, CIOMS Guidelines, and the International Conference on Harmonization of Technical Requirements for Registration of Pharmaceuticals for Human Use—Good Clinical Practice (ICH-GCP), reviewed and approved the study (Approval no. S029h/66_Exp). Given that the study utilized secondary data, the requirement for documenting informed consent was waived, and this waiver was authorized by the Institutional Review Board of the Royal Thai Army Medical Department.

### 5.2. Experimentally Envenomed Rat Studies

The procedure regarding animal experiments was performed as previously described [22,23].

#### 5.2.1. Snake Venoms

Specimens of Eastern Russell’s viper (*D. siamensis*) were maintained in captivity at QSMI, the Thai Red Cross Society Bangkok, Thailand. Venom was extracted from several snakes (>20 specimens, male and female), pooled, and then frozen before being freeze-dried. Venoms were weighed and reconstituted in phosphate-buffered saline (PBS), and venom protein concentrations were measured using a BCA protein assay (Pierce Biotechnology, Rockford, IL, USA).

#### 5.2.2. Animal Ethics and Care

Male Sprague-Dawley rats were purchased from Nomura-Siam International Co. Ltd., Bangkok, Thailand. Rats were housed in stainless steel containers with food and drinking water ad libitum access. Approvals for all experimental procedures were obtained from the Subcommittee for Multidisciplinary Laboratory and Animal Usage of Phramongkutklao College of Medicine and the Institutional Review Board, Royal Thai Army Department, Bangkok, Thailand (Documentary Proof of Ethical Clearance no: IRBRTA 1130/2560) in accordance with the U.K. Animal (Scientific Procedure) Act, 1986, and the National Institutes of Health guide for the care and use of laboratory animals (NIH Publications No. 8023, revised 1978) [22].

#### 5.2.3. Anesthetized Rat Preparation

Male Sprague-Dawley rats weighing 300–350 g were anesthetized using Zoletil^®^ (20 mg/kg, Virbac, Texas, USA) and Xylazine^®^ (5 mg/kg, Kepro, Woerden, The Netherlands) via the intraperitoneal (i.p.) route. The additional anesthetic was administered throughout the experiment as required. A midline incision was made in the cervical region, and cannulae were inserted into the right jugular vein (for drug administration), the carotid artery (for measurement of blood pressure), and the trachea (for artificial respiration if required). Arterial blood pressure was recorded using a reusable pressure transducer filled with heparinized saline (25 U/mL). Systemic blood pressure was monitored on a MacLab system (ADInstruments, Bella Vista, Australia). The rats were kept under a heat lamp during the experiment. Normal saline (100 µL) was administered via the right jugular vein to maintain blood volume if required. At the conclusion of the experiment, animals were euthanized by an overdose of anesthetic agent (i.v.) [22,23].

#### 5.2.4. Venom Dose Optimization

Preliminary experiments examined the nephrotoxic effects of *D. siamensis* venom in anesthetized rats via intramuscular (i.m.) doses of 50 µg/kg (i.e., 15 µg/300 g), 100 µg/kg (i.e., 30 µg/300 g), and 200 µg/kg (i.e., 60 µg/300 g). Venom was dissolved in 0.9% NaCl and administered using a 27-gauge needle into the extensor muscles of the right hind limb. Venom doses < 200 µg/kg failed to induce a morphological change in heart, liver, and kidney within 3 h. Subsequently, the dose of 200 µg/kg (i.m.) was chosen to induce toxicity in this study.

### 5.3. Measurement of Serum D. siamensis Venom Concentration

All methods in this section were performed as previously described [47].

#### 5.3.1. Preparation of IgG

The saturated ammonium sulfate was used for immunoglobulin (IgG) separation. Hyperimmune horse plasma against Russell’s viper venom was obtained from the horse farm of QSMI (Queen Saovabha Memorial Institute). An amount of 30 mL of horse plasma was precipitated with ammonium sulfate at 40% saturation. The mixture was stirred for 30 min and incubated overnight in a cold room (4 °C). The mixture was then centrifuged at 8000× *g* rpm (Eppendorf 5810R) for 10 min, and the precipitate was dissolved in 30 mL of PBS buffer (0.01M phosphate buffer pH 7.2 containing 0.15 N NaCl). The solution was made 33% saturated with ammonium sulfate and stirred for 30 min. After centrifuging at 8000× *g* rpm for 10 min, the precipitate was dissolved in 20 mL of PBS buffer. Finally, the solution was dialyzed 3 times against the PBS buffer.

#### 5.3.2. Preparation of Peroxidase-Conjugated Specific IgG of Russell’s Viper Snake Venom

Peroxidase enzyme was used to label the venom-specific horse IgG. Briefly, 10 mg of peroxidase enzyme was dissolved in 2 mL of 0.3 M sodium bicarbonate buffer pH 8.1, then 0.2 mL of 1%DNFB (2,4-Dinitrofluorobenzene) in ethanol was added, stirring for 1 h at room temperature. Then, 2 mL of 0.06 M sodium metaperiodate was added and stirred for 1 h. Two milliliters of 0.16 M ethylene glycol were added and stirred for 1 h. The mixture was dialyzed 3 times with 0.01 M sodium carbonate buffer pH 9.5 at 4 °C. The 10 mg of specific horse IgG was dissolved in 1 mL of 0.01 M sodium carbonate buffer pH 9. 5, added into the prepared mixture, and stirred for 2–3 h at room temperature. Then, 5 mg of sodium borohydride was added to the mixture by stirring for 3 h at 4 °C and dialyzed in PBS pH 7.2 at 4 °C. The mixtures were separated by Sephadex G-200 gel filtration chromatography. The column (1.6 × 95 cm) was packed with Sephadex G-200 (Pharmacia, Uppsala, Sweden) and eluted with PBS pH 7.2 at the flow rate of 14 mL/h. The eluate was collected in 1 mL fractions, and the absorbance was measured at 280 and 403 nm [47].

#### 5.3.3. Detection of Russell’s Viper Snake Venom by ELISA Method

Plates were coated with 50 µL/well of 0.05 M carbonate buffer pH 9.6 containing horse monovalent antivenom (1:2000) and incubated for 3 h at 37 °C. After washing three times with 0.01M PBS pH 7.0, the plate was blocked with 1% skim milk in PBS for 1 h and washed with PBS. A sample or serial dilution of snake venom was added to the plate (50 μL/well) and incubated for 1 h at room temperature. After washing, peroxidase-conjugated specific horse IgG was added to the plate and incubated for 1 h. After washing with PBS, substrate (1,2-phenylenediamine dihydrochloride; OPD) was added to the plate and incubated for 30 min; 0.5 M sulfuric acid was used as the stopping solution. The absorbance was measured at 492 nm using an ELISA plate reader. The concentration of samples was calculated from a standard plot using a polynomial parameter [47].

### 5.4. Histopathological Studies

#### 5.4.1. Histological Preparation for Hematoxylin and Eosin (H&E) Staining

Histopathological examination of the heart, liver, and kidney of envenomed animals was determined following previously described methods [24]. At the conclusion of the in vivo test (i.e., 3 and 6 h), rats were sacrificed (for the survivors), and the liver, heart, and both kidneys were removed and preserved in 10% formalin. All tissues were dehydrated in graded ethanol series through 70, 80, 90, 95, and 100% with two changes for 1 h each. Three washings of xylene, for 30 min each, were then completed before embedding the tissues in paraffin. Embedded samples were cross-sectionally cut and stained with H&E. Tissues were examined and photographed under an Olympus light microscope (BX-50, Olympus, Tokyo, Japan) [48].

#### 5.4.2. Histological Preparation for Transmission Electron Microscopy Method

Pieces of liver, heart, and kidney tissues (~1 mm^3^) were immediately fixed in 2.5% buffered glutaraldehyde. The specimens were post-fixed in 1% osmium tetroxide, dehydrated, infiltrated with propylene oxide and embedded in resin. Semi-thin sections about 0.5 µm or 1.0 µm were stained with Toluidine blue, used as a guideline to the area of interest and further trimmed. Ultrathin sections of about 60 nm were cut on an ultramicrotome. These were stained with uranyl acetate and lead citrate. Ultrathin sections were spread mostly on 200 or 300-mesh copper grids and stained with uranyl acetate and lead citrate solutions. The sections were examined and photographed using a transmission electron microscope (TEM-JEM2010, JEOL Ltd., Tokyo, Japan). The degree of morphological changes was determined by previously described criteria [48].

### 5.5. Statistical Analysis

For a clinical retrospective study, baseline characteristics were examined using descriptive statistics. Continuous data were presented as either mean with standard deviation (SD) or median with interquartile range (IQR) as appropriate, while categorical data were expressed as frequency and percentage.

All graphical representations and experimental statistical analyses were performed using the PRISM 8 (GraphPad Software, San Diego, CA, USA) software package. The intramuscular venom concentration versus time data were fitted to both mono-exponential (one compartment) and bi-exponential (two compartment) models using PRISM, and a comparison was made to determine which was a better fit; 95% confidence intervals (95%CI) were calculated in PRISM.

## Figures and Tables

**Table 1:** Demographic characteristics of patients following *D. siamensis* bites in Pattananikom Hospital in Central Thailand.

Characteristics	*n* (%)
**Total**	23 (100.0)
**Year**	
2014–2017	4 (17.4)
2018–2020	6 (26.1)
2021–2023	13 (56.5)
**Sex**	
Male	19 (82.6)
Female	4 (17.4)
**Admission**	
Outpatient	0 (0.0)
Admit	6 (26.1)
Refer	17 (73.9)
**Bitten area**	
Trunk	0 (0.0)
Upper extremities	6 (26.1)
Lower extremities	17 (73.9)
**Clinical Manifestation**	
Local Pain	15 (65.2)
Swelling	18 (78.3)
Erythema	20 (87.0)
Compartment syndrome	0 (0.0)
Dyspnea	1 (4.4)
Ptosis	0 (0.0)
**Duration from the bite to arrival at the hospital**	
≤15 min	4 (17.4)
15–60 min	11 (47.8)
60–90 min	5 (21.7)

**Table 2:** Blood chemistry analysis and white blood cell count following *D. siamensis* envenoming (** missing data were found during the initial examination due to not being recorded).

Blood Chemistry and White Blood Cell Count (Normal Range)	*n* (%)
**Serum sodium (135–145 mmol/L)**	
No hyponatremia (≥135 mmol/L)	18 (78.3)
Hyponatremia (<135 mmol/L)	5 (21.7)
**Serum potassium (3.5–5.0 mmol/L)**	
No hypokalemia (≥3.5 mmol/L)	8 (34.8)
Hypokalemia (<3.5 mmol/L)	15 (65.2)
**Serum bicarbonate (22–31 mmol/L)**	
No metabolic acidosis (≥22 mmol/L)	15 (65.2)
Metabolic acidosis (<22 mmol/L)	8 (34.8)
** Creatinine level (0.7–1.2 mg/dL)	
0.7–1.2 mg/dL	19 (86.4)
≥1.2 mg/dL	3 (13.6)
**White blood cell count (4 × 10^3^ – 11 × 10^3^** **cells/µL)**	
<4 × 10^3^ cells/µL	-
4–11 × 10^3^ cells/µL	19 (82.6)
>11 × 10^3^ cells/µL	4 (17.4)
** Chloride (90– **105 mmol/L)**	
<90 mmol/L	-
90–105 mmol/L	15 (65.22)
>105 mmol/L	4 (17.39)

**Table 3:** Hematologic characteristics following *D. siamensis* bites and antivenom treatment.

Systemic Effects	*n* (% of 23 Patients)
**VCT (min)**	
≤20	23 (100)
>20	-
**INR**	
≤1.2	13 (56.5)
>1.2	6 (26.1)
**Platelet (×10^9^/L)**	
Mean ± SD	202.0 ± 83.1
<140 × 10^3^	5 (21.7)
≥140 × 10^3^	18 (78.3)
**Systemic bleeding**	
No	4 (17.4)
Yes	19 (82.6)
**Local effects (Pain, swelling, redness)**	
No	3 (13.0)
Yes	20 (87.0)
**Antivenom treatment**	
No	13 (56.5)
Yes	10 (43.5)

## Data Availability

The datasets generated during and/or analyzed during the current study are available from the corresponding author on reasonable request.
